# An evaluation of vascular anomalies and incidental findings in patients with Turner syndrome

**DOI:** 10.1186/1532-429X-14-S1-P130

**Published:** 2012-02-01

**Authors:** Alisa Kanfi, Michael O'Loughlin, Sean M Lang, Olga H Toro-Salazar

**Affiliations:** 1 Radiology, Hartford Hospital, Hartford, CT, USA; 2 Pediatric Cardiology, Connecticut Children's Medical Center, Hartford, CT, USA; 3 Pediatric Cardiology, Yale New Haven Children's Hospital, New Haven, CT, USA

## Summary

Multiple thoracic vascular anomalies have been described in the setting of Turner syndrome. Careful evaluation of both venous and arterial anatomy in the chest is prudent when evaluating these cases. By highlighting the less commonly associated vascular malformations in Turner syndrome through key images, we intend to raise awareness and familiarity of these potentially clinically significant anomalies. We also hope to reinforce the importance of evaluating extra-cardiac structures to uncover the additional incidental but significant findings.

## Description of clinical presentation

Prior studies have demonstrated a high prevalence of congenital cardiovascular malformations in Turner syndrome patients. Classic associations with bicuspid aortic valves and coarctation of the aorta are well known, but with the development of 3D MR angiography, additional vascular malformations have been highlighted. Patients with Turner syndrome have increased associations with aortic arch anomalies, including bovine arch configurations and aberrant right subclavian artery origins. Additionally, venous drainage malformations such as partial anomalous pulmonary venous return (PAPVR) and persistent left-sided superior vena cava (SVC) have been identified.

## Diagnostic techniques and their most important findings

We reviewed a total of 29 cardiac Magnetic Resonance Imaging (MRI) exams performed on twenty patients with Turner syndrome. The studies were performed on General Electric HDX 1.5 Tesla MRI machines with gadolinium enhanced magnetic resonance angiograms (MRA) of the thoracic vessels. Image post-processing was performed using a General Electric Advanced Windows workstation.

Several vascular anomalies were noted: Aortic coarctation (30%; 6 patients), PAPVR (15%, 3 patients), bicuspid aortic valves (45%, 9 patients), persistent left sided SVCs, (25%, 5 patients), and aberrant right subclavian arteries (15%, 3 patients). One patient had an aortic arch origin of the left vertebral artery. Two patients demonstrated two right renal arteries. Additional incidental MR findings, such a hepatic hemangioma and a thyroid mass were also seen in two patients.

## Learning points from this case

Turner syndrome is commonly associated with bicuspid aortic valves and coarctation of the aorta. However, additional vascular anomalies are commonly seen including: PAPVR, persistent left-sided SVC, aberrant right subclavian artery, and bovine aortic arch configuration. Recognition of the prevalence and classic MR imaging appearance of the additional anomalies will hopefully improve detection on imaging and subsequently aid in patient care and medical/surgical treatment planning. Additional thorough evaluation of the cardiac MRI can highlight important extra-cardiac and non-vascular findings.

**Figure 1:**
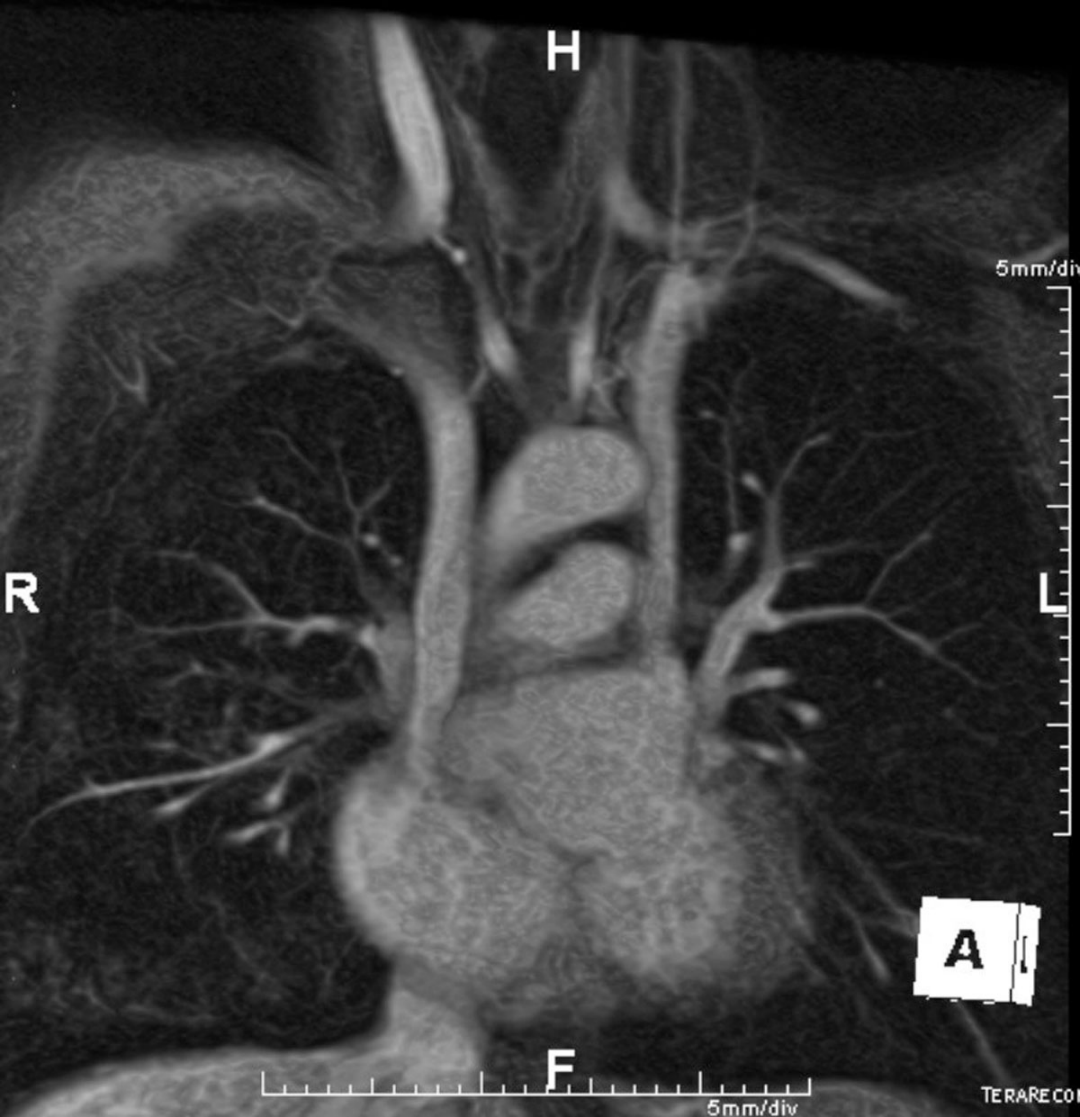
Contrast-enhanced 3D MR angiogram shows a persistent left superior vena cava, which drains into the coronary sinus.

**Figure 2:**
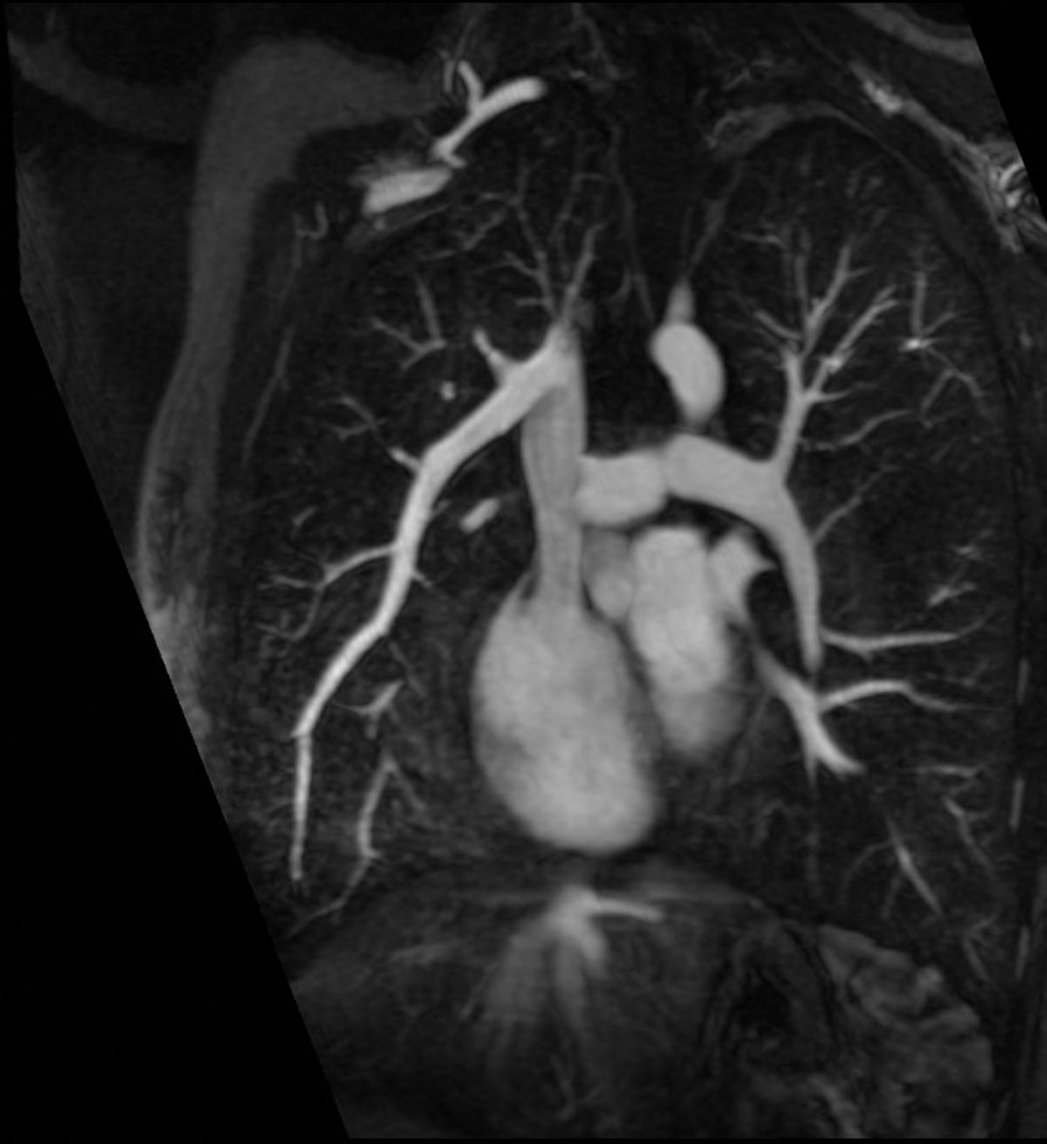
Contrast-enhanced MR angiogram demonstrates partial anomalous pulmonary venous return. A pulmonary vein draining the right upper and middle lobes terminates in the superior vena cava.

